# Plasma Levels of Food-Derived Metabolites as Biomarkers of Parkinson’s Disease

**DOI:** 10.3390/ijms27010016

**Published:** 2025-12-19

**Authors:** Xiaoxue Dong, Yilong Zheng, Evelyn Ting Ying Tan, Qiao Yang Sun, Bin Xiao, Eng King Tan, Yun-Cheng Wu, Zhi Dong Zhou

**Affiliations:** 1 Department of Neurology, Shanghai General Hospital, Shanghai Jiao Tong University School of Medicine, No.86, Wujin Road, Shanghai 200080, China; xiaoxuedong21@gmail.com (X.D.); yunchw@medmail.com.cn (Y.-C.W.); 2 Department of Neurology, National Neuroscience Institute of Singapore, 11 Jalan Tan Tock Seng, Singapore 308433, Singapore; qiaoyangsun001@gmail.com (Q.Y.S.); xiao.bin@singhealth.com.sg (B.X.); 3 SingHealth PGY1 Program, Singapore General Hospital, Outram Road, Singapore 169608, Singapore; yilongzheng@u.nus.edu; 4 School of Biological Science, Nanyang Technological University, 50 Nanyang Ave, Singapore 639798, Singapore; tty.evelyn8150@gmail.com; 5 Signature Research Program in Neuroscience and Behavioral Disorders, Duke-NUS Graduate Medical School Singapore, 8 College Road, Singapore 169857, Singapore; 6 Department of Neurology, Singapore General Hospital, Outram Road, Singapore 169608, Singapore

**Keywords:** Parkinson’s disease, biomarkers, food-derived metabolites, caffeine, paraxanthine, trigonelline, piperine, sitosteryl hexoside, *LRRK2* mutations, *GBA1* mutations, PPMI cohort, disease progression

## Abstract

Parkinson’s disease (PD) is a progressive neurodegenerative disorder shaped by genetic factors such as *LRRK2* and *GBA1* mutations, as well as dietary and metabolic influences. Food-derived plasma metabolites—including caffeine, paraxanthine, trigonelline, piperine, and sitosteryl hexoside—have emerged as promising, accessible biomarkers for early detection, progression monitoring, and therapeutic targeting, yet their longitudinal behavior and genetic interactions remain insufficiently characterized. Using the Parkinson’s Progression Markers Initiative (PPMI) cohort (*n* = 455; 303 PD patients, 152 controls), we quantified plasma levels of these metabolites by quantitative LC-MS/MS with batch correction, examining sporadic PD and genetically defined subgroups (*LRRK2*-PD [PDL], *GBA1*-PD [PDG], dual-mutation PD [PDGL], and prodromal equivalents). Baseline one-way ANOVA showed significantly lower caffeine and paraxanthine in PDL (*p* = 0.0467, *p* = 0.0178) and PDG (*p* = 0.0408), reduced piperine in PDL (*p* = 0.0009), PDG (*p* = 0.0257), and prodromal *LRRK2* (*p* = 0.0168), and elevated sitosteryl hexoside in PDG (*p* = 0.0184). Longitudinal regression analyses revealed that in sporadic PD, caffeine negatively correlated with MDS-UPDRS parts I (β = −2, *p* = 0.0475) and III (β = −7.2, *p* = 0.007), trigonelline declined over time and was inversely associated with part III (β = −1.7, *p* = 0.0069), and sitosteryl hexoside negatively correlated with parts II (β = −68.3, *p* = 0.042) and III (β = −74.1, *p* = 0.0425). In PDL, sitosteryl hexoside inversely correlated with part I (β = −54.2, *p* = 0.0049), while in PDGL, paraxanthine showed negative associations with part II (β = −18.5, *p* = 0.00327). These findings demonstrate subgroup-specific alterations in food-derived metabolites and consistent inverse associations with PD severity, supporting their potential as non-invasive biomarkers, particularly in *LRRK2/GBA1* mutation carriers, and highlighting the need for longitudinal validation and dietary intervention trials to advance personalized PD management.

## 1. Introduction

Parkinson’s disease (PD) is a progressive neurodegenerative disorder characterized by the loss of dopaminergic neurons in the substantia nigra, leading to motor impairments such as bradykinesia, rigidity, and tremor, alongside non-motor symptoms including cognitive decline [1], affecting millions worldwide with an increasing prevalence in aging populations [2,3]. The identification of reliable biomarkers for PD is crucial for early diagnosis, monitoring disease progression, and evaluating therapeutic interventions [4]. Recent research has highlighted the potential role of food-derived metabolites as accessible plasma biomarkers, which may reflect dietary influences, metabolic alterations, or interactions with genetic factors implicated in PD pathogenesis [5,6].

Emerging evidence highlights the influence of dietary and lifestyle factors on PD risk and progression, particularly through food-derived compounds that may exert neuroprotective effects. Caffeine and its metabolites like paraxanthine have been associated with reduced PD risk and altered plasma levels in affected individuals, potentially linked to block adenosine receptors and anti-inflammatory properties [5,7,8]. Similarly, trigonelline, a coffee-derived alkaloid, shows promise in mitigating neurological deficits in PD models [9]. Piperine, an alkaloid from black pepper, has demonstrated neuroprotective properties in PD models by attenuating motor and cognitive impairments [10], and plant sterols like sitosteryl hexoside may possess the anti-inflammatory effects in neurodegenerative contexts [11]. Genetic variants, including pathogenic mutations in leucine-rich repeat kinase 2 (*LRRK2*) [12] and glucocerebrosidase beta 1 (*GBA1*) genes [13], further modulate PD risk and progression, with emerging evidence linking them to metabolic dysregulations in caffeine and other pathways [14,15,16], but there remains limited evidence supporting their ability to predict disease progression [17].

Longitudinal cohorts like the Parkinson’s Progression Markers Initiative (PPMI) offer critical insights into these dynamics by providing comprehensive biospecimen data for biomarker validation [18]. Our current investigation utilizes data from the PPMI cohort, specifically employing quantitative liquid chromatography-tandem mass spectrometry (LC-MS/MS) data on plasma levels of five food-based biomarkers, including caffeine, paraxanthine, trigonelline, piperine and sitosteryl hexoside. We aimed to first compare these biomarker levels across cohorts of healthy individuals and those diagnosed with PD, with subgroup analyses based on the presence or absence of pathogenic mutations in the *LRRK2* and *GBA1* genes. Additionally, we investigated the temporal trends exhibited by these biomarkers over time and explored their potential associations with disease severity, aiming to uncover insights into their role as indicators of PD progression and therapeutic targets.

## 2. Results

A total of 455 participants were enrolled in the study, comprising 303 individuals diagnosed with PD and 152 healthy controls. Among the PD patients, 114 (37.6%) belonged to the sporadic PD subgroup, 118 (38.9%) to the PDL subgroup, 67 (22.1%) to the PDG subgroup, and 4 (1.3%) to the PDGL subgroup, respectively. Within the healthy control group, 47 (30.9%) were categorized as healthy controls, 69 (45.4%) as belonging to the ProdL subgroup, 33 (21.7%) as belonging to the ProdG subgroup, and 3 (2.0%) as belonging to the ProdGL subgroups. The baseline ages of the respective study cohorts are presented in Supplementary Table S1.

**Figure 1:**
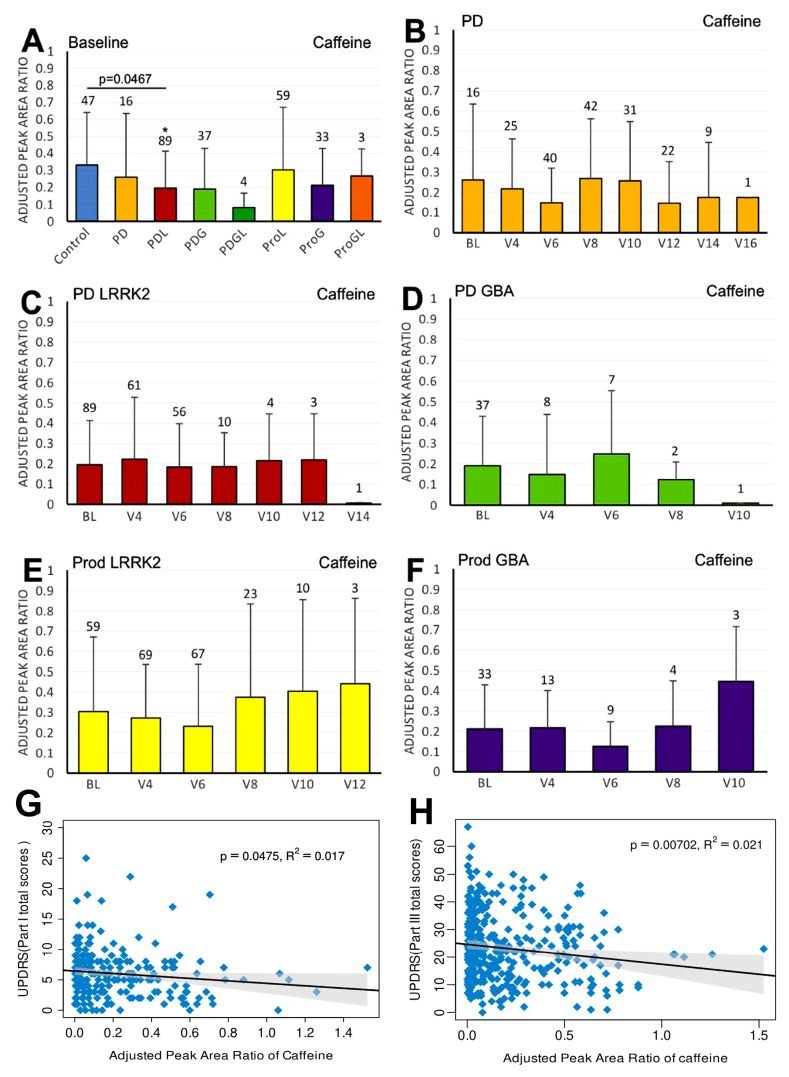
LC/MS analysis of plasma caffeine levels and their association with MDS-UPDRS scores on linear regression. (**A**) Baseline plasma caffeine levels across groups. Comparisons versus healthy controls were performed using one-way ANOVA. (**B**–**F**) Longitudinal changes in caffeine levels in (**B**) PD without pathogenic *LRRK2*/*GBA1* mutations, (**C**) PD with *LRRK2* mutations, (**D**) PD with *GBA1* mutations, (**E**) prodromal *LRRK2*, and (**F**) prodromal *GBA1* subjects. Baseline versus follow-up time points were compared using one-way ANOVA. Numbers of participants are indicated above columns. Data are shown as mean ± SD; no statistical testing was conducted when *n* < 5; * *p* < 0.05 versus healthy controls (**A**) or baseline (**B**–**F**). (**G**,**H**) Linear regression of plasma caffeine levels against MDS-UPDRS scores in the PD population: (**G**) Part I and (**H**) Part III. Regression lines include 95% confidence intervals (shaded). Pearson R and *p*-values are shown; *p* < 0.05 was considered statistically significant.

**Figure 2:**
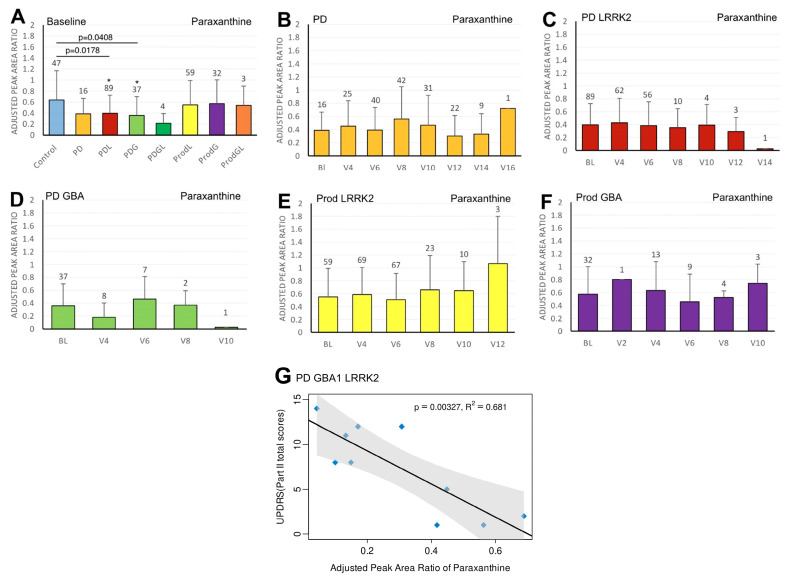
LC/MS analysis of plasma paraxanthine levels and its association with MDS-UPDRS scores on linear regression. (**A**) Baseline plasma paraxanthine levels across groups. Comparisons versus healthy controls were performed using one-way ANOVA. (**B**–**F**) Longitudinal changes in paraxanthine levels in (**B**) PD patients without pathogenic *LRRK2*/*GBA1* mutations, (**C**) PD with *LRRK2* mutations, (**D**) PD with *GBA1* mutations, (**E**) prodromal *LRRK2*, and (**F**) prodromal *GBA1* subjects. Baseline versus follow-up time points were compared using one-way ANOVA. Numbers of participants are indicated above each column. Data are shown as mean ± SD; no statistical testing was conducted when *n* < 5; * *p* < 0.05 versus healthy controls (**A**) or baseline (**B**–**F**). (**G**) Linear regression of plasma paraxanthine levels against MDS-UPDRS Part II scores in the PDGL population. Regression lines include 95% confidence intervals (shaded). Pearson correlation coefficient (R) and *p*-value are reported; *p* < 0.05 was considered statistically significant.

**Figure 3:**
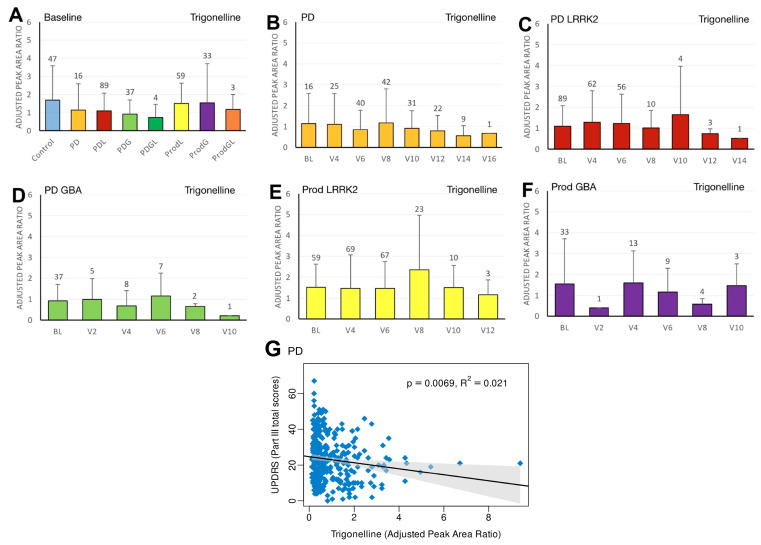
LC/MS analysis of plasma trigonelline levels and its association with MDS-UPDRS scores on linear regression. (**A**) Baseline plasma trigonelline levels across groups. Comparisons versus healthy controls were performed using one-way ANOVA. (**B**–**F**) Longitudinal changes in trigonelline levels in (**B**) PD patients without pathogenic *LRRK2*/*GBA1* mutations, (**C**) PD patients with *LRRK2* mutations, (**D**) PD patients with *GBA1* mutations, (**E**) prodromal *LRRK2* subjects, and (**F**) prodromal *GBA1* subjects. Baseline versus follow-up time points were compared using one-way ANOVA. Numbers of participants are indicated above each column. Data are shown as mean ± SD; no statistical testing was conducted when *n* < 5. (**G**) Linear regression of plasma trigonelline levels against MDS-UPDRS Part III scores in the PD population. Regression lines include 95% confidence intervals (shaded). Pearson correlation coefficient (R) and *p*-value are reported; *p* < 0.05 was considered statistically significant.

**Figure 4:**
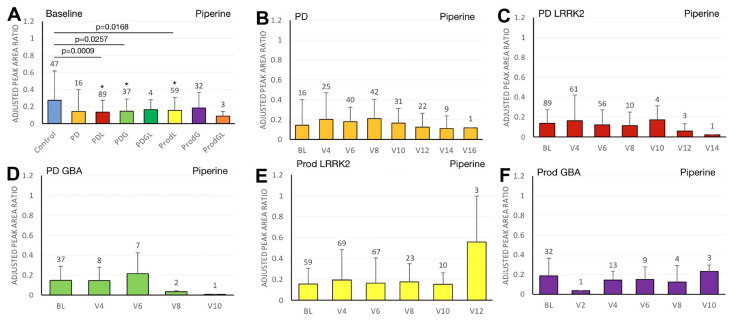
LC/MS analysis of plasma piperine levels and its association with MDS-UPDRS scores on linear regression. (**A**) Baseline plasma piperine levels across groups. Comparisons versus healthy controls were performed using one-way ANOVA. (**B**–**F**) Longitudinal changes in piperine levels in (**B**) PD patients without pathogenic *LRRK2*/*GBA1* mutations, (**C**) PD patients with *LRRK2* mutations, (**D**) PD patients with *GBA1* mutations, (**E**) prodromal *LRRK2* subjects, and (**F**) prodromal *GBA1* subjects. Baseline versus follow-up time points were compared using one-way ANOVA. Numbers of participants are indicated above each column. Data are shown as mean ± SD; no statistical testing was conducted when *n* < 5; * *p* < 0.05 versus healthy controls (**A**) or baseline (**B**–**F**).

**Figure 5:**
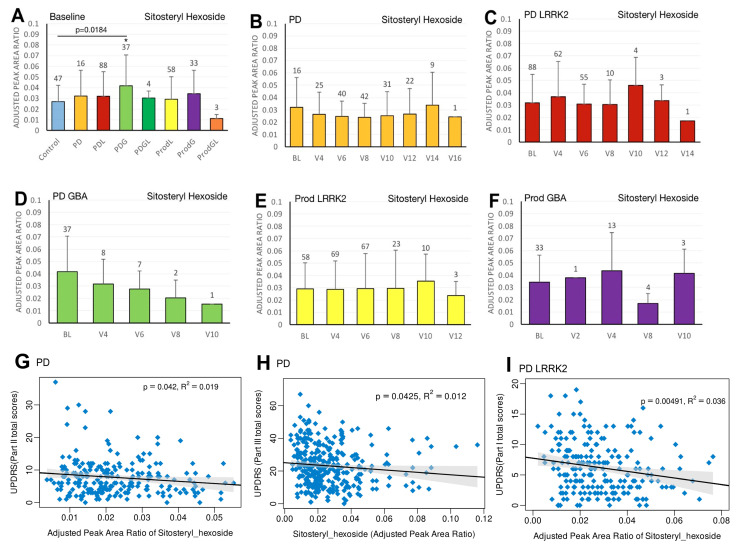
LC/MS analysis of plasma sitosteryl hexoside levels and its association with MDS-UPDRS scores on linear regression. (**A**) Baseline plasma sitosteryl hexoside levels across groups. Comparisons versus healthy controls were performed using one-way ANOVA. (**B**–**F**) Longitudinal changes in sitosteryl hexoside levels in (**B**) PD patients without pathogenic *LRRK2*/*GBA1* mutations, (**C**) PD patients with *LRRK2* mutations, (**D**) PD patients with *GBA1* mutations, (**E**) prodromal *LRRK2* subjects, and (**F**) prodromal *GBA1* subjects. Baseline versus follow-up time points were compared using one-way ANOVA. Numbers of participants are indicated above each column. Data are shown as mean ± SD; no statistical testing was conducted when *n* < 5; * *p* < 0.05 versus healthy controls (**A**) or baseline (**B**–**F**). (**G**–**I**) Linear regression analyses of plasma sitosteryl hexoside levels against MDS-UPDRS scores: (**G**) Part II and (**H**) Part III in the PD population, and (**I**) Part I in the PDL population. Regression lines include 95% confidence intervals (shaded). Pearson correlation coefficients (R) and *p*-values are reported; *p* < 0.05 was considered statistically significant.

We first compared the differences in plasma biomarker levels between healthy controls and the various PD subgroups. We found that the PDL cohort had significantly lower baseline caffeine levels than healthy controls (*p* = 0.0467) (Figure 1A). Similarly, levels of baseline paraxanthine, a caffeine metabolite, were significantly lower than those of healthy controls within both the PDL (*p* = 0.0178) and PDG (*p* = 0.0408) cohorts (Figure 2A). However, baseline plasma levels of trigonelline did not differ significantly between healthy controls and any of the PD subgroups (all *p* > 0.05) (Figure 3A). In addition, baseline levels of piperine were significantly lower than those of healthy controls in the PDL (*p* = 0.0009), PDG (*p* = 0.0257), and ProdL (*p* = 0.0168) cohorts (Figure 4A). Conversely, baseline levels of sitosteryl hexoside were significantly higher than those of healthy controls within the PDG cohort (*p* = 0.0184) (Figure 5A).

We subsequently analyzed the trend in the biomarker levels over time and the associations of biomarker levels with disease severity, using regression models with 95% confidence intervals, supplemented by correlation analyses and generalized additive models (GAMs) in Supplementary Table S2.

In the sporadic PD cohort, a significant negative correlation was seen between caffeine and MDS-UPDRS parts I (beta = −2; 95% CI = −4, 0; *p* = 0.0475) (Figure 1G) and III (beta = −7.2; 95% CI = −12.5, −2; *p* = 0.007) (Figure 1H). Moreover, we observed a declining trend in trigonelline levels over time (Figure 3B), and this trend was supported by linear regression analysis, which revealed a significant negative association between trigonelline and MDS-UPDRS part III total score (beta = −1.7; 95% CI = −2.9, −0.5; *p* = 0.0069) (Figure 3G). There was also a significant negative association between sitosteryl hexoside and MDS-UPDRS parts II (beta = −68.3; 95% CI = −134, −2.5; *p* = 0.042) (Figure 5G) and III (beta = −74.1; 95% CI = −145.6, −2.5; *p* = 0.0425) (Figure 5H).

In the PDL cohort, we found a significant negative correlation between sitosteryl hexoside and MDS-UPDRS part I (beta = −54.2; 95% CI = −91.8, −16.6; *p* = 0.0049) (Figure 5I).

In the PDGL cohort, we found significant negative correlations between paraxanthine and MDS-UPDRS part II (beta = −18.5; 95% CI = −28.9, −8.2; *p* = 0.00327) (Figure 2G).

## 3. Discussion

The PPMI cohort serves as a vital resource for elucidating the role of food-derived biomarkers in PD pathogenesis and progression, offering longitudinal biospecimen data for robust metabolomic analyses. By employing quantitative LC-MS/MS to quantify plasma levels of caffeine, paraxanthine, trigonelline, piperine, and sitosteryl hexoside, our study delineates differences across healthy controls and PD subgroups, including those with *LRRK2* and *GBA1* mutations, while exploring temporal dynamics and correlations with disease severity. These dietary metabolites, sourced from everyday consumables like coffee, tea, pepper, and plant sterols, may reflect metabolic perturbations in PD, potentially serving as non-invasive indicators of resistance, progression, or therapeutic response. This aligns with recent metabolomics efforts identifying plasma signatures for early PD detection and genetic stratification.

We selected the five food-derived plasma metabolites based on converging evidence from epidemiology, mechanistic studies, human metabolomics, biomarker feasibility, and the availability of high-quality longitudinal measurements in the PPMI dataset. These metabolites are consistently implicated in PD risk and neuroprotection, particularly caffeine and paraxanthine, which have well-established effects on dopaminergic signaling, neuroinflammation, and mitochondrial function [8,19]. Trigonelline and piperine demonstrate antioxidant, anti-inflammatory, and dopaminergic rescue effects in preclinical PD models [9,10,20], while sitosteryl hexoside, a phytosterol glycoside, has emerging relevance to neuroinflammation and cholesterol-associated pathways implicated in neurodegeneration [11,21,22,23,24]. Multiple human metabolomics studies report reduced levels of caffeine-related metabolites and trigonelline in PD patients and in *LRRK2* mutation carriers [14,25]. Alterations in piperine and plant sterol derivatives have also been observed in PD metabolic signatures, suggesting potential relevance to early metabolic dysfunction. Furthermore, GBA1-related lysosomal impairment may intersect with phytosterol metabolism and autophagy-modulating metabolites such as paraxanthine and sitosteryl hexoside [15,26]. Collectively, these biological and methodological considerations provide a strong rationale for focusing on these metabolites when evaluating early disease processes and gene–diet interactions in PD. In addition, their stability, suitability for LC–MS/MS quantification, and availability across multiple PPMI timepoints make them particularly robust candidates for biomarker assessment.

### 3.1. Caffeine

The motor benefits of caffeine have been well-documented in both animal and human studies of PD, and accumulating evidence indicates that its neuroprotective actions arise through multiple convergent molecular pathways [5,27,28,29]. The primary mechanism is antagonism of the adenosine A2A receptor (A2AR), which is highly enriched in striatopallidal neurons and tightly coupled to dopaminergic signaling. By blocking A2AR, caffeine reduces excessive GABAergic output from the indirect pathway, enhances D2 receptor signaling, and preserves dopaminergic neurotransmission [30,31,32]. At the cellular level, A2AR inhibition attenuates glutamatergic excitotoxicity, stabilizes mitochondrial membrane potential, and decreases intracellular Ca^2+^ overload—all key contributors to dopaminergic neuron vulnerability [30,33]. Furthermore, A2AR blockade suppresses microglial activation and downstream NF-κB signaling, thereby reducing neuroinflammation and pro-inflammatory cytokine release [30,34]. In parallel, caffeine enhances mitochondrial biogenesis through PGC-1α activation, promotes autophagic clearance of damaged proteins, and diminishes oxidative stress via Nrf2-dependent antioxidant pathways, mechanisms that are particularly relevant in PD pathogenesis [33,35,36].

Notably, prior metabolomic analyses have identified diminished caffeine and caffeine-metabolite levels as biomarkers of increased PD vulnerability [7,25,37,38], with effects more pronounced in *LRRK2*-associated PD than in idiopathic cases [14,16,39]. This aligns with our findings showing reduced baseline plasma caffeine in the PD-*LRRK2* (PDL) cohort, suggesting that habitual caffeine intake may exert heightened neuroprotection in genetically predisposed individuals. Emerging preclinical evidence further indicates a functional interaction between caffeine and LRRK2 signaling: A2AR antagonism may be particularly effective in mitigating the pathobiology of LRRK2 kinase-enhanced α-synuclein [14,16,40], but still requiring targeted cellular and animal studies. Consistent with this mechanistic link, our regression analyses revealed a negative correlation between caffeine’s adjusted peak area ratio and UPDRS scores, reinforcing the association between higher caffeine exposure and improved motor status, in line with recent salivary caffeine profiling in PD patients [29]. Together, these converging lines of evidence support the hypothesis that caffeine may delay PD onset or slow progression, particularly in *LRRK2* mutation carriers. Nonetheless, the long-term clinical efficacy of caffeine as a biomarker of PD remains to be validated in large, prospective, and genotype-stratified trials.

### 3.2. Paraxanthine

Paraxanthine, the major metabolite of caffeine, recapitulates many of caffeine’s neuroprotective properties while also engaging additional molecular targets that may be especially relevant to dopaminergic neuron resilience [7,19,25,41]. Like caffeine, paraxanthine functions as a non-selective adenosine receptor antagonist with appreciable activity at the A2AR—an effect that reduces indirect-pathway overactivity in the basal ganglia, preserves D2 receptor signaling, and thereby supports striatal dopaminergic tone and motor function [42,43]. Beyond adenosinergic blockade, paraxanthine has been shown to engage ryanodine receptor (RyR) channels to modulate intracellular Ca^2+^ signaling in dopaminergic neurons; controlled RyR stimulation can activate adaptive calcium-dependent signaling pathways that strengthen mitochondrial function and promote survival signaling [19]. Paraxanthine’s actions also converge on cellular stress pathways: preclinical work indicates reductions in oxidative stress markers and enhancement of antioxidant responses (including downstream Nrf2-axis activity), as well as facilitation of mitochondrial biogenesis and autophagic flux—mechanisms that reduce accumulation of dysfunctional mitochondria and misfolded proteins implicated in PD [44].

Importantly for genetic subtypes, metabolomic studies have repeatedly reported lower circulating levels of caffeine and its metabolites (including paraxanthine) in PD patients and in carriers of pathogenic *LRRK2* variants, supporting a link between impaired xanthine metabolism or altered exposure and disease susceptibility [7,14,45]. Our finding of significantly reduced paraxanthine at baseline in PD-*LRRK2* and PD-*GBA1* cohorts therefore aligns with these metabolomic signatures and suggests that diminished paraxanthine exposure—or accelerated clearance—may associate with elevated risk or earlier phenoconversion in these genotypes. Also, our regression analyses—showing an inverse relationship between paraxanthine adjusted peak area ratio and MDS-UPDRS in the PD-*GBA1*-*LRRK2* (PDGL) subgroup—are consistent with an association between higher paraxanthine exposure and milder motor impairment. However, direct mechanistic links between paraxanthine and GBA1-related pathobiology are less well characterized. *GBA1* mutations principally perturb lysosomal glucocerebrosidase activity, lysosomal clearance, and α-synuclein homeostasis; given paraxanthine’s reported promotion of autophagy and lysosomal competence in other models, it is biologically plausible that paraxanthine could attenuate GBA1-related vulnerability, but empirical data are sparse and warrant investigation in polygenic and genotype-specific PD models.

Taken together, preclinical mechanistic data and human metabolomics support a preventive role for paraxanthine in PD, particularly in genetically predisposed populations; nonetheless, it requires randomized, genotype-stratified clinical trials and focused mechanistic work (e.g., paraxanthine treatment in LRRK2 and GBA1 cellular/animal models) to validate paraxanthine as a therapeutic candidate or biomarker.

### 3.3. Trigonelline

Trigonelline, a niacin-derived coffee metabolite, likely confers neuroprotection through several convergent mechanisms relevant to PD. It appears to preserve nigrostriatal neurons by supporting dopaminergic survival pathways and attenuating excitotoxicity [9,46,47]; it reduces oxidative stress via upregulation of endogenous antioxidant responses (e.g., Nrf2-linked effectors) and by improving mitochondrial function and bioenergetics (enhancing mitochondrial biogenesis and stabilizing membrane potential) [48,49]. Trigonelline also inhibits neuroinflammation by limiting microglial activation and downstream NF-κB signaling, and may facilitate proteostasis through modest promotion of autophagy/lysosomal clearance—actions that together reduce vulnerability to α-synuclein and mitochondrial insults [9,48,49]. Our observation of lower baseline trigonelline across PD groups and its inverse correlation with MDS-UPDRS in the PD cohort is consistent with metabolomic reports that link reduced trigonelline to increased PD risk [14,45] and may reflect altered niacin metabolism or reduced coffee exposure. These data nominate trigonelline as a candidate severity biomarker and biological modifier; targeted longitudinal studies and controlled supplementation trials in genotype-stratified cohorts are warranted.

### 3.4. Piperine

Piperine, the principal alkaloid of black pepper, has shown neuroprotective potential in PD animal models, protecting against nigrostriatal dopaminergic loss, alpha-synuclein aggregation, autophagy, oxidative damage, and neuroinflammation while enhancing motor and cognitive functions [10,20,50,51,52]. Mechanistically, several studies report that piperine stimulates autophagic flux (via P2RX4 activation and suppression of PI3K/AKT/mTOR signaling in rodent PD models), thereby enhancing clearance of aggregated α-synuclein and reducing proteotoxic stress in substantia nigra neurons [51,52,53]. Concomitantly, piperine exerts robust antioxidant and mitochondrial-protective effects: it lowers lipid peroxidation and mitochondrial ROS, preserves mitochondrial membrane potential and electron-transport activity, and sustains ATP production—actions that blunt intrinsic apoptotic cascades in vulnerable dopaminergic cells [54]. Piperine also suppresses neuroinflammatory pathways central to PD progression. In microglial and in vivo inflammatory models, piperine downregulates TLR4/NF-κB signaling, lowers expression of iNOS/COX-2 and pro-inflammatory cytokines (TNF-α, IL-1β), and increases anti-inflammatory mediators—attenuating inflammation-driven neuronal injury that accelerates α-synuclein toxicity [10,20,55]. Beyond these direct neuronal and glial actions, piperine influences systemic and gut–brain axes implicated in PD. Recent work indicates that piperine modulates gut microbiota composition and can enhance gut–brain autophagy-mediated α-synuclein clearance, an effect relevant to prodromal/pathogenic propagation theories of PD [51].

Though preliminary and derived from animal models, these effects merit human translational investigation. In our findings, baseline reductions in piperine levels in PDL, PD-*GBA1* (PDG), and prodromal *LRRK2* (ProdL) groups versus controls underscore its neuroprotective promise in these cohorts, consistent with preclinical evidence, but the conclusion still requires longitudinal sampling, controlled dietary metadata, and mechanistic studies in genotype-specific PD models. Our results advocate for prospective cohorts to solidify piperine as a prognostic biomarker and evaluate its supplementation for PD progression modulation.

### 3.5. Sitosteryl Hexoside

Sitosteryl hexoside, a phytosterol glycoside commonly found in plant-based foods, has a complex and potentially bidirectional relationship with neurodegeneration, and current studies are still limited and inconsistent [11,56,57,58,59,60]. Phytosterols classically exert antioxidant, anti-inflammatory and membrane-stabilizing effects: they can scavenge free radicals, preserve membrane fluidity and lipid raft integrity, and modulate nuclear receptors (e.g., PPARs) and downstream transcriptional programs that reduce oxidative stress and inflammatory signaling [11,21,22,23,24], which provide a biologically plausible mechanism for protection of vulnerable nigrostriatal neurons against PD-relevant damage. Our regression results—negative correlations between circulating sitosteryl hexoside and UPDRS scores in PD and PD-LRRK2 cohorts—are consistent with a protective interpretation in which higher phytosterol glycoside levels associate with milder motor severity, possibly via the antioxidant/membrane-stabilizing effects of phytosterols.

However, the biology of sitosteryl hexoside is not uniformly protective. A specific phytosterol glycoside, β-sitosterol β-D-glucoside (BSSG), has been used experimentally to induce progressive parkinsonism in rodents—promoting α-synuclein aggregation, nigrostriatal degeneration, neuroinflammation, and reproducible motor deficits—thereby functioning as a neurotoxicant in several models and providing a widely used BSSG PD model [61,62]. Though replication across labs is variable [63], the model is still sufficient to interpret that not all sterol glycosides are uniformly beneficial.

In the setting of intact lysosomal/autophagic function, phytosterols may integrate into membranes and support lipid homeostasis and signaling that protect neurons; in contrast, when lysosomal processing is compromised (as in *GBA1* deficiency) or when exposure to certain glycosides is excessive or delivered centrally, sterol glycosides may accumulate as poorly cleared substrates that seed proteostatic stress and promote α-synuclein aggregation [64]. This mechanistic framework helps interpret our baseline elevation of sitosteryl hexoside in GBA1-mutated PD versus controls. *GBA1* mutations perturb lysosomal glucocerebrosidase activity and lipid metabolism, leading to accumulation of glucosylsphingosine and broader sphingolipid/sterol dysregulation [26]; impaired lysosomal clearance could therefore increase circulating or tissue levels of sterol glycosides or alter their metabolic fate, potentially converting a normally benign or protective molecule into one that exacerbates proteostatic stress and α-synuclein pathology [64,65,66]. Thus, elevated sitosteryl hexoside in *GBA1* carriers may reflect lysosomal dysfunction and altered sterol handling rather than a protective signal.

Taken together, the evidence for sitosteryl hexoside as a PD biomarker or therapeutic effector is intriguing but inconclusive. Key implications are: (1) circulating sitosteryl hexoside associations with motor severity may be protective in some contexts (PD, PD-*LRRK2*) yet pathological in others (*GBA1*) depending on lysosomal competence and sterol species; (2) functional studies in genotype-stratified models (*GBA1* and *LRRK2* cellular and animal systems) are essential to determine whether manipulation of sitosteryl hexoside (or modulation of sterol metabolism) will be beneficial or harmful. Therefore, further studies across genetic subtypes are required before considering sitosteryl hexoside as a therapeutic agent—while its potential as a biomarker for subtype-specific lipid dysregulation and lysosomal impairment remains promising but requires validation in larger, longitudinal and mechanistically informed cohorts.

### 3.6. Practical Clinical Recommendations

Although our findings require prospective validation, several pragmatic clinical considerations emerge regarding dietary metabolites as potential modifiers or biomarkers of Parkinson’s disease. First, regular caffeine consumption—within generally accepted safe limits (≈200–400 mg/day)—may confer neuroprotective benefit, particularly in individuals with heightened genetic susceptibility such as *LRRK2* mutation carriers, who consistently show reduced caffeine and metabolite levels. Clinicians may reasonably encourage moderate coffee or tea intake in caffeine-tolerant individuals without contraindications (e.g., severe arrhythmia, uncontrolled hypertension, pregnancy), while recognizing that caffeine is not yet an established disease-modifying therapy. Given the strong A2A receptor–linked mechanisms and epidemiologic support, incorporating longitudinal caffeine/metabolite measurement into routine follow-up in research settings may help stratify risk or monitor progression.

Second, emerging evidence suggests that paraxanthine, as the primary active metabolite of caffeine, may be equally or more informative as a biomarker of dopaminergic vulnerability. Although supplementation is not currently available clinically, paraxanthine levels may be a useful exploratory marker in patients with *GBA1* or *LRRK2* mutations, where reduced exposure associates with worse motor status. Clinicians managing genetically at-risk individuals may consider participation in research studies incorporating metabolomic profiling to refine risk assessment.

Third, coffee-derived trigonelline and pepper-derived piperine show consistent inverse associations with PD risk or severity in our data and prior metabolomic studies. While there is insufficient evidence to recommend targeted supplementation, encouraging dietary patterns rich in polyphenol- and alkaloid-containing foods (e.g., coffee, black pepper, plant-based diets) may support overall neuroprotective nutrient intake. These compounds appear safe in typical dietary amounts and may contribute to a broader lifestyle-based risk-reduction strategy.

Finally, sitosteryl hexoside and related phytosterol glycosides require a more nuanced clinical interpretation. In idiopathic PD and *LRRK2*-PD, higher circulating levels may reflect protective antioxidant and membrane-stabilizing actions, whereas in *GBA1*-related PD, elevated levels may instead signal lysosomal dysfunction and aberrant sterol handling. Thus, phytosterol supplementation should not be recommended for PD patients—particularly *GBA1* mutation carriers—until genotype-specific safety and mechanistic studies are completed. Clinicians should also be aware that sterol-enriched supplements, marketed for lipid lowering, may have uncertain implications in PD and should be used cautiously.

Overall, while these dietary metabolites show promise as non-invasive biomarkers and potential lifestyle-modifiable factors, clinical implementation should proceed conservatively. Integration of metabolomic profiling into genotype-stratified research protocols, coupled with personalized dietary counseling, may represent the most immediate translational avenue until interventional trials establish efficacy and safety across PD subtypes.

### 3.7. Strengths and Future Directions

This study harnesses the robust framework of the PPMI, a landmark longitudinal cohort that provides comprehensive, standardized biospecimen and clinical data, enabling detailed biomarker validation and progression tracking in PD. Key strengths include the within-participant design, which allows simultaneous assessment of multiple biomarkers, enhancing the reliability of associations between plasma metabolomics and clinical outcomes such as MDS-UPDRS scores. The inclusion of genetic subgroups (*LRRK2* and *GBA1* mutations) facilitates stratified analyses, revealing mutation-specific metabolic patterns, such as reduced caffeine and paraxanthine levels in *LRRK2* carriers, which align with recent findings on gene-environment interactions in PD.

Despite these strengths, several limitations must be acknowledged. The observational design of PPMI precludes establishing causality between biomarker levels and PD outcomes, as associations may be influenced by reverse causation or unmeasured confounders such as dietary habits and lifestyle factors. Plasma metabolomics, while accessible, may not fully capture brain-specific metabolic changes, and variability in sample collection or processing could introduce bias, a common challenge in omics-based PD biomarker research. Subgroup sample sizes—particularly for rare genetic combinations such as *GBA1–LRRK2*—may limit statistical power and generalizability. To address this limitation and maintain statistical rigor, we adopted a threshold of *n* < 5 as a criterion for excluding any inferential statistical testing. This threshold is consistent with widely accepted statistical practice, as groups with fewer than five observations lack sufficient degrees of freedom for reliable variance estimation, are highly sensitive to outliers, and yield unstable or uninterpretable *p*-values. These measures ensure conservative, statistically sound interpretations while reflecting the constraints of the PPMI dataset and enabling longitudinal assessment of food-derived metabolites across PD genetic subgroups. Additionally, while LC–MS/MS offers high sensitivity, metabolomics data can be affected by batch effects, analytical variability, and difficulties in distinguishing causative from associative changes in cross-sectional comparisons. Future research should incorporate randomized interventions, larger and more diverse cohorts, and integrated multi-omics approaches to address these gaps and further validate these biomarkers for clinical use.

## 4. Materials and Methods

### 4.1. Study Design

The PPMI is an observational, international, multicenter cohort study aiming to identify biomarkers of PD progression with longitudinal follow-up (https://www.ppmi-info.org/, accessed on 5 December 2025). Details regarding the PPMI study have been previously published [18,67].

Our study compares the levels of various plasma biomarkers between subgroups in our cohort at baseline. We also analyzed the trend of the biomarkers from the initial baseline visit (BL) to subsequent follow-up appointments at different time points at V02 (6 months), V04 (12 months), V05 (18 months), V06 (24 months), V08 (36 months), V10 (48 months), V12 (60 months), V14 (84 months), and V16 (108 months). Associations between levels of the plasma biomarkers and disease severity were also explored.

We selected five food-derived plasma metabolites—caffeine, paraxanthine, trigonelline, piperine, and sitosteryl hexoside—based on converging evidence from epidemiology, mechanistic studies, human metabolomics, biomarker feasibility, and the availability of high-quality longitudinal data in the PPMI cohort. Cohort subgroups analyzed include healthy controls, healthy subjects with pathogenic *LRRK2* gene mutations (prodromal *LRRK2* (ProdL)), *GBA1* mutations (prodromal *GBA1* (ProdG)), and both pathogenic *LRRK2* and *GBA1* gene mutations (prodromal *LRRK2* and *GBA1* (ProdGL)), PD subjects with no pathogenic *LRRK2* or *GBA1* gene mutations (PD), and PD subjects with pathogenic *LRRK2* gene mutations (PDL), *GBA1* mutations (PDG), and both pathogenic *LRRK2* and *GBA1* gene mutations (PDGL).

### 4.2. LC/MS Protocol

Plasma samples were thawed on ice and centrifuged at 3500× *g* for 10 min at 4 °C. A 10 µL aliquot was transferred to a 96-well Agilent Captiva collection plate (Agilent Technologies, Santa Clara, CA, USA) and processed using the Agilent Bravo Metabolomics Sample Prep Platform following the Bravo Low Volume Plasma Metabolite/Lipid protocols. Proteins were precipitated by adding 112.5 µL of 1:1 (*v*/*v*) methanol/ethanol containing internal standards. The plate was shaken at 1000 rpm for 1 min and incubated at room temperature for 10 min. Ultrapure water (82.5 µL) was added, followed by shaking at 500 rpm for 1 min and incubation at room temperature for 10 min. A 200 µL aliquot was transferred to an Agilent Captiva EMR-Lipid 96-well plate for filtration. The plate was washed twice with 250 µL of 1:1:1 (*v*/*v*/*v*) water/ethanol/methanol. The metabolite filtrate was dried under N_2_ for 4 h and reconstituted in 200 µL LC-MS-grade methanol (Sigma-Aldrich, St. Louis, MO, USA). Lipids were eluted with 1.8 mL of 1:2 (*v*/*v*) dichloromethane/methanol, collected in a glass-coated 96-well microplate (Waters Corporation, Milford, MA, USA), dried under N_2_ for 4 h, and reconstituted in 200 µL methanol.

LC-MS targeted analysis of metabolites and lipids was performed on an Agilent Infinity II 1290 UHPLC system (Agilent Technologies, Santa Clara, CA, USA) coupled to a QTRAP 6500+ triple quadrupole mass spectrometer (SCIEX, Framingham, MA, USA) operated in positive electrospray ionization (ESI+) mode. Samples (5 µL) were injected; data acquired in multiple reaction monitoring (MRM) mode (transitions/energies in Table 1) and quantified with non-endogenous internal standards (Table 1), external calibration for lipids (0.01–10 µM), using MultiQuant software (version 3.0.2; SCIEX, Framingham, MA, USA). For metabolites: chromatographic separation was achieved using a Waters ACQUITY UPLC BEH Amide column (1.7 µm, 2.1 × 150 mm; Waters Corporation, Milford, MA, USA) maintained at 40 °C with a flow rate of 0.40 mL/min. Mobile phases: A (water + 10 mM ammonium formate + 0.1% formic acid), B (acetonitrile + 0.1% formic acid). Gradient: 0–1 min, 95% B; 1–7 min, to 50% B; 7–7.1 min, to 95% B; 7.1–10 min, 95% B. ESI settings: curtain gas 30 psi, collision gas medium, ion spray 5500 V, temp 600 °C, gas 1 50 psi, gas 2 60 psi, entrance potential 10 V, collision exit 12.5 V. For lipids: separation was performed using a Waters ACQUITY UPLC BEH C18 column (1.7 µm, 2.1 × 100 mm; Waters Corporation, Milford, MA, USA) maintained at 55 °C with a flow rate of 0.25 mL/min, with external calibration (0.01–10 µM). Mobile phases: A (60:40 acetonitrile/water + 10 mM ammonium formate + 0.1% formic acid), B (90:10 isopropanol/acetonitrile + 10 mM ammonium formate + 0.1% formic acid). Gradient: 0–8 min, 45–99% B; 8–9 min, 99% B; 9–9.1 min, to 45% B; 9.1–10 min, 45% B. ESI settings: curtain gas 40 psi, collision gas medium, ion spray 5500 V, temp 250 °C, gas 1 55 psi, gas 2 60 psi, entrance potential 10 V, collision exit 12.5 V.

### 4.3. Plasma Biomarker Quantification

This study analyzed five different batches of samples using both targeted and untargeted metabolomics and lipidomics methods. The samples in each batch were selected such that independent factors and covariates of interest were randomized and evenly distributed between batches. Additionally, three pooled control and three pooled PD case plasma samples were distributed evenly within and between batches. Each of the pooled samples was injected 3–6 times within each batch, and values from replicate extractions and injections were used to calculate assay CV and inter-batch deviations. Missing peak areas were identified and visualized using the naniar package, and missing values were imputed using the k-nearest neighbors imputation method. Following data imputation, areas for endogenous metabolites and lipids were divided by areas of analyte-specific spiked surrogate stable isotope internal standards. Subsequently, inter-batch variances of area ratios were corrected by using the Combat function provided in the sva package. Relative log expression plots of area ratios were plotted to visualize variations within and across batches. Following correction for between-batch effects, imputed values were removed. For each non-imputed quantified analyte, unnormalized peak area, ratios of endogenous metabolite area to surrogate internal standards, and batch-adjusted area ratios were reported. Batch-adjusted area ratios are recommended for downstream analysis.

### 4.4. LRRK2 and GBA Genotyping

Genetic testing was carried out by the genetic testing core. Before and following testing, individuals who were carriers but not yet showing symptoms received genetic counseling from certified genetic counselors affiliated with the University of Indiana or trained on-site personnel. The genetic analysis for *LRRK2* focused on detecting pathogenic mutations *G2019S* and *R1441G*. On the other hand, *GBA1* genetic testing included screening for the *N370S* mutation in all participants, with a subset also undergoing testing for additional mutations such as *L483P*, *L444P*, *IVS2+1*, and *84GG*.

### 4.5. Statistical Analysis

All statistical analyses were performed using R software (version 4.2.2; R Foundation for Statistical Computing, Vienna, Austria). Prior to analysis, outlier biomarker values were removed using the 1.5 interquartile range (IQR) rule, excluding observations below Q1 − 1.5 × IQR or above Q3 + 1.5 × IQR. Records missing biomarker or MDS-UPDRS total score data were excluded.

For baseline comparisons, the mean and standard deviation (SD) of adjusted peak area ratios were plotted for each subgroup. Group differences were evaluated using one-way analysis of variance (ANOVA). Following a significant ANOVA result, Dunnett’s post hoc test was applied to perform pairwise comparisons against the healthy control group (Supplementary Table S2). Normality of the biomarker distributions was assessed using the Shapiro–Wilk test, while homogeneity of variances across groups was evaluated using Levene’s test (Supplementary Table S2). Although baseline data demonstrated deviations from normality, variance homogeneity was satisfied, and ANOVA was considered robust under these conditions.

For longitudinal analyses, the mean (SD) adjusted peak area ratios across all available time points were plotted for the PD, PDL, PDG, ProdG, and ProdGL subgroups. Pairwise comparisons between baseline and subsequent time points were conducted only when the sample size at the comparison time point was ≥5. Time points with fewer than five observations were presented descriptively without inferential testing. This threshold was adopted to maintain statistical rigor, as groups with fewer than five observations have insufficient degrees of freedom for reliable variance estimation and yield unstable or uninterpretable *p*-values. When assumptions of normality or homoscedasticity were not met, non-parametric alternatives were considered; however, ANOVA remained appropriate given the relatively large sample sizes and equal variances in the primary analyses. *p*-values are reported only for comparisons that met the minimum sample size requirement (*n* ≥ 5) and reached statistical significance.

Associations between biomarker levels and MDS-UPDRS total scores (Parts I–IV) were examined using univariate linear regression. For biomarker–UPDRS pairs exhibiting non-normal distributions, data characteristics were explicitly evaluated to justify model selection, and potential non-linear relationships were further explored using Generalized Additive Models (GAMs). Regression analyses were performed independently within each subgroup (PD, PDL, PDG, and PDGL). Pearson correlation coefficients were calculated for normally distributed data, while Spearman correlation was applied for non-normal distributions (Supplementary Table S2).

A 95% confidence interval (CI) for each regression line was constructed using the predict() function in R with interval = “confidence”. Predicted values were generated across a sequence of evenly spaced biomarker values, yielding estimated mean responses with upper and lower CI bounds. These intervals were visualized as shaded ribbons surrounding the regression lines to reflect the uncertainty of the estimated relationships. A two-sided significance threshold of *p* < 0.050 was applied for all analyses.

## 5. Conclusions

This study demonstrates that food-derived plasma metabolites are significantly altered in Parkinson’s disease and show subtype-specific associations with clinical severity, particularly in carriers of *LRRK2* and *GBA1* mutations. Using the PPMI cohort, we identified consistent inverse relationships between caffeine, paraxanthine, trigonelline, piperine, and sitosteryl hexoside levels and MDS-UPDRS scores, supporting their potential utility as non-invasive biomarkers. These findings highlight the importance of gene–environment–metabolite interactions in PD and support further longitudinal validation and interventional studies to advance precision medicine approaches in PD.

## Figures and Tables

**Table 1:** Metabolomics/lipidomics in positive mode parameters.

Name	Internal Standard	Q1 *m*/*z*	Q3 *m*/*z*	CE (V)	% Samples with Missing Area Ratio
Caffeine	Niacinamide-d4	195.1	138	20	0.1
Paraxanthine	Niacinamide-d4	181.1	124	24	0.1
Trigonelline	Methionine-d3	138.1	94.1	20	0
Piperine	Niacinamide-d4	286.1	201.1	20	0.1
Sitosteryl hexoside	CE (18:1(d7))	594.6	397.4	17	0.1

The percent of samples with missing area ratio is calculated across the entire set of samples, including the pooled control samples.

## Data Availability

The data presented in this study are available on request from the corresponding author. Processed metabolomic datasets generated from PPMI samples and all analysis scripts used in this work can be provided upon reasonable request. Raw clinical and biospecimen data were obtained from the Parkinson’s Progression Markers Initiative (PPMI) and are publicly accessible to registered users at the PPMI data repository (https://www.ppmi-info.org/, accessed on 5 December 2025) in accordance with PPMI data-use policies. No additional proprietary or restricted datasets were generated in this study.

## Supplementary Materials

The following supporting information can be downloaded at: https://www.mdpi.com/article/10.3390/ijms27010016/s1.
