# Supplementary material for: Plasma Levels of Food-Derived Metabolites as Biomarkers of Parkinson’s Disease
Source: Int J Mol Sci. 2025 Dec 19;27(1):16. doi: 10.3390/ijms27010016 (PMC12785599; doi:10.3390/ijms27010016)
Supplement: Supplementary file 1 [file ijms-27-00016-s001.zip › Supplementary Table S1.pdf]

Supplementary Table S1: Baseline age of respective study cohorts

Cohort	Baseline age: mean (standard deviation)
Total (n=455)	63.4 (8.7)
Control (n=47)	64.6 (9.1)
PD (n=114)	60.7 (8.4)
PDL (n=118)	64.1 (8.4)
PDG (n=67)	63.0 (8.9)
PDGL (n=4)	59.0 (10.8)
ProdL (n=69)	63.3 (7.3)
ProdG (n=33)	65.8 (5.1)
ProdGL (n=3)	70.1 (7.7)
